# CT-based deep learning integrating pancreatic imaging and body composition for severity prediction in acute pancreatitis

**DOI:** 10.1186/s13244-026-02443-w

**Published:** 2026-10-28

**Authors:** Dan Zeng, Meng He, Bingbing Lin, Yu Liu, Xinwei Wang, Jie Lu

**Affiliations:** 1 Department of Radiology and Nuclear Medicine, Xuanwu Hospital Capital Medical University, Beijing, China; 2 Beijing Key Laboratory of Magnetic Resonance Imaging and Brain Informatics, Beijing, China; 3 Department of Radiology, Chongqing General Hospital, Chongqing, China

**Keywords:** Pancreatitis, Deep learning, Body composition, Tomography (X-ray computed)

## Abstract

**Objectives:**

To develop and validate a deep learning (DL)-based CT model integrating pancreatic imaging and CT-derived body composition for severity prediction in acute pancreatitis (AP).

**Materials and methods:**

In this retrospective dual-center study, patients with AP who underwent CT within 72 h of symptom onset were enrolled from January 2021 to September 2025 at Center 1 and January 2023 to September 2024 at Center 2. The primary dataset (*n* = 426) was randomly divided into a training set (*n* = 298) and an internal test set (*n* = 128), while 110 patients from Center 2 served as the external test set. A three-dimensional DL model was developed for pancreatic imaging, and two-dimensional DL models were constructed for L3-level visceral adipose tissue (VAT) and skeletal muscle (SM). Six predictive models were constructed: clinical, pancreas, VAT, SM, pancreas-clinical (PC), and combined. Performance was evaluated using receiver operating characteristic analysis, DeLong testing, calibration, decision curve analysis, net reclassification improvement (NRI), and integrated discrimination improvement (IDI).

**Results:**

The combined model achieved area under the curve (AUCs) of 0.897 (95% CI: 0.862–0.932), 0.909 (95% CI: 0.861–0.958), and 0.910 (95% CI: 0.856–0.964) in the training, internal test, and external test sets, respectively. The combined model outperformed the PC model in the training set (*p* = 0.002) and external test set (*p* = 0.020). NRI and IDI analyses confirmed the incremental predictive value of body composition features.

**Conclusions:**

A CT-based DL model integrating pancreatic imaging, body composition, and clinical variables demonstrated good performance for AP severity prediction.

**Key Points:**

***Question*** Early severity stratification in AP remains challenging because conventional clinical and imaging assessments may not fully capture pancreatic injury and systemic disease status.

***Findings*** A CT-based DL model integrating pancreatic imaging, body composition, and clinical variables demonstrated good performance for severity prediction in AP.

***Critical Relevance Statement*** This study evaluates the value of integrating pancreatic imaging and CT-derived body composition information and supports the use of routinely acquired CT data for early risk stratification, potentially facilitating more individualized monitoring and management of AP.

**Graphical Abstract:**

**Figure:**
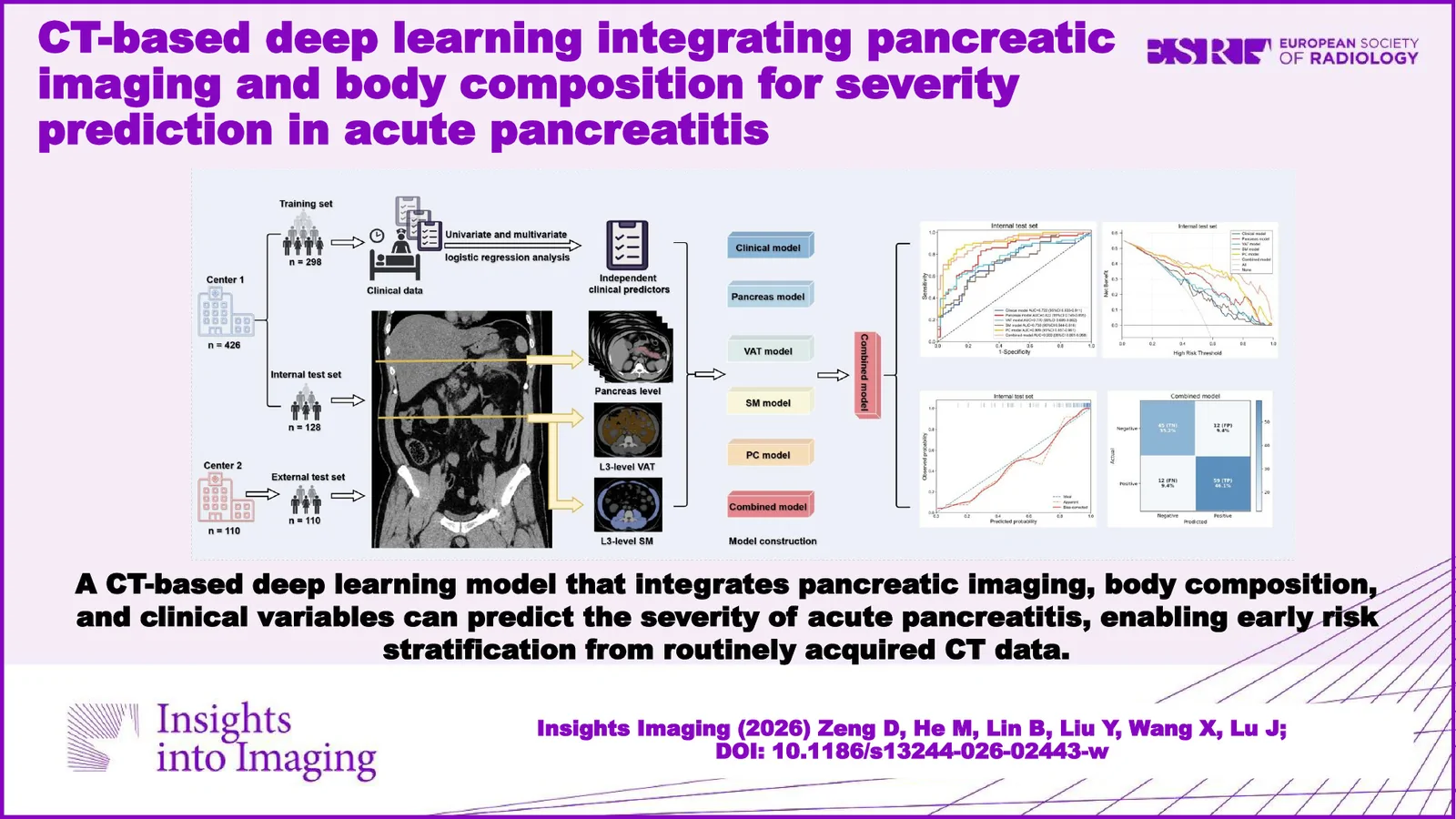

## Introduction

Acute pancreatitis (AP) is a common gastrointestinal inflammatory disease with highly variable clinical outcomes [1]. Although most patients experience a mild and self-limited disease course, approximately 15%–20% progress to moderately severe acute pancreatitis (MSAP) or severe acute pancreatitis (SAP), which are associated with persistent organ failure, local complications, prolonged hospitalization, and increased mortality [2]. Early identification of patients at risk of severe disease is therefore essential for optimizing clinical management and guiding monitoring strategies.

Several clinical scoring systems have been developed for AP severity prediction, including the Ranson score, acute physiology and chronic health evaluation II (APACHE II), and bedside index for severity in acute pancreatitis (BISAP). However, many of these tools require multiple clinical variables and relatively complex calculations, which limit their clinical usability for early risk stratification [3–5]. Imaging-based scoring systems such as the computed tomography severity index (CTSI) and modified CTSI rely primarily on morphologic assessment of pancreatic necrosis and local complications using contrast-enhanced CT. Because these morphologic changes may not be fully established during the first 72 h after symptom onset, the utility of conventional CT-based scoring systems for early risk stratification may be limited [6].

AP involves not only pancreatic injury but also peripancreatic fat involvement and systemic inflammatory responses, both of which contribute to disease progression [7]. CT-derived body composition may thus provide complementary imaging information beyond pancreatic morphology. Previous studies have indicated that visceral adipose tissue (VAT) and skeletal muscle (SM) are associated with AP severity and prognosis [8–14]. Because abdominal computed tomography (CT) is routinely performed in patients with AP, these body composition metrics can be obtained through opportunistic assessment without additional imaging. However, prior studies have largely relied on manual measurements or simple area- and density-based parameters, which may not adequately capture complex imaging patterns or spatial heterogeneity. Therefore, more advanced automated quantitative approaches are needed to better characterize CT-derived body composition associated with disease severity.

Recent advances in artificial intelligence, particularly deep learning (DL), have enabled automated extraction of complex imaging features beyond conventional quantitative measurements. Several studies have demonstrated that CT-based DL models can improve risk prediction in AP by capturing spatial patterns and imaging heterogeneity that may not be detectable using traditional approaches [15–17]. However, most existing DL studies in AP have primarily focused on pancreatic imaging features alone, while systemic factors such as body composition, which may reflect host inflammatory response and physiological reserve, remain relatively underexplored. Moreover, the potential incremental value of integrating pancreatic imaging features with CT-derived body composition information has not been fully investigated. Therefore, this study aimed to develop and validate a DL-based CT model for severity prediction in AP by integrating pancreatic imaging, CT-derived body composition, and clinical variables.

## Materials and methods

### Patients

This retrospective dual-center study was approved by the Ethics Committees of Xuanwu Hospital Capital Medical University (2020-092) and Chongqing General Hospital (KY S2024-070-01), and the requirement for informed consent was waived due to the retrospective design. A total of 536 consecutive patients with AP were retrospectively enrolled from two tertiary centers. In Center 1, 531 consecutive patients diagnosed with AP between January 2021 and September 2025 were screened, and 426 were included in the primary dataset, which was randomly divided into a training set (*n* = 298) and an internal test set (*n* = 128). In Center 2, 178 consecutive patients diagnosed with AP between January 2023, and September 2024 were screened, and 110 were included as the external test set. Data partitioning was performed at the patient level, with no patient overlap among the training, internal test, and external test sets.

**Fig. 1:**
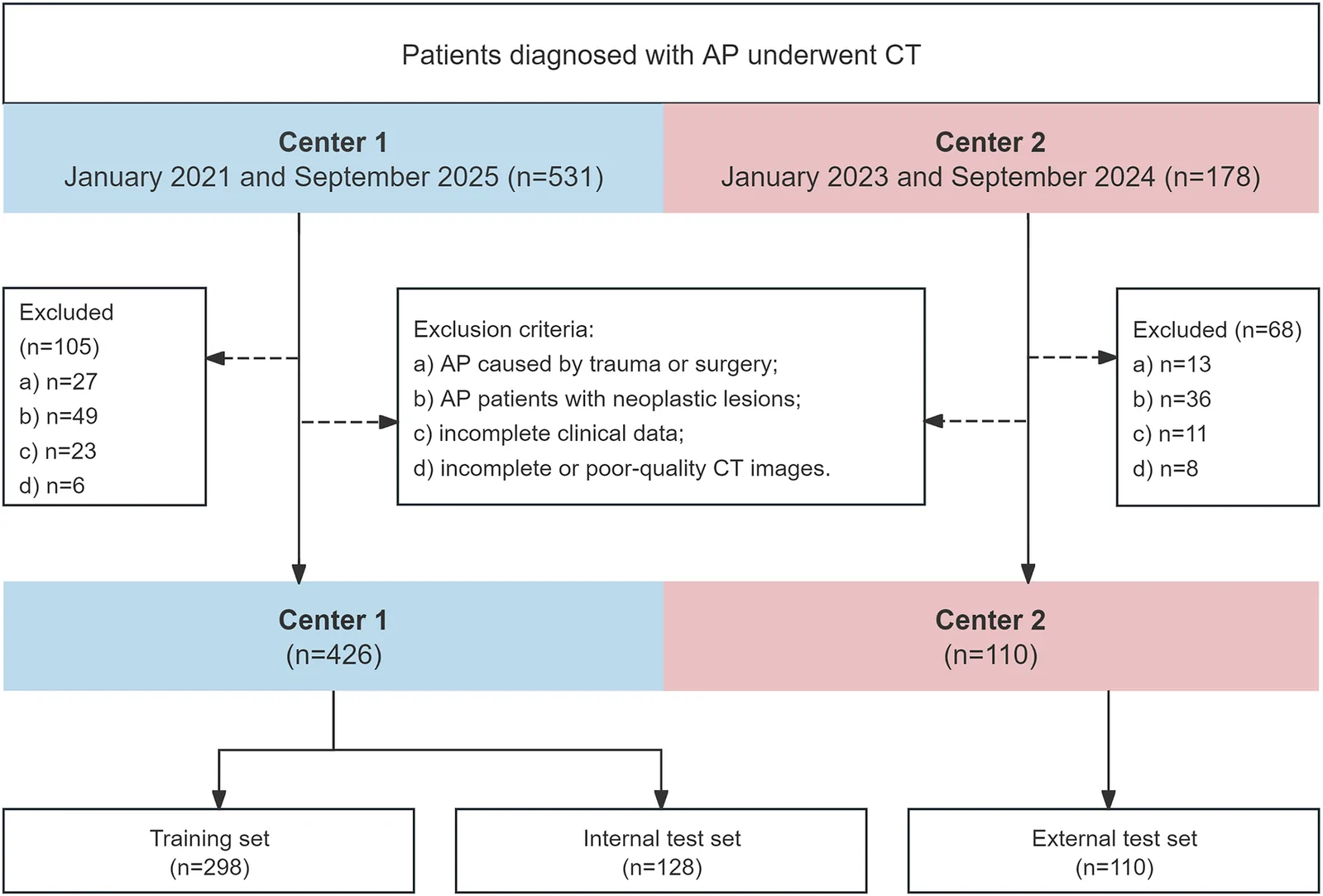
Patient selection flowchart of the study population

The same inclusion and exclusion criteria were applied in both centers. Inclusion criteria were: (a) first episode of AP; (b) abdominal CT within 72 h after symptom onset; and (c) age ≥ 18 years. Exclusion criteria were: (a) AP caused by trauma or surgery; (b) AP with neoplastic lesions; (c) incomplete clinical data; and (d) incomplete or poor-quality CT images. AP was diagnosed according to established diagnostic criteria (Appendix S1). The enrollment flowchart is shown in Fig. 1. This study was reported in accordance with the Checklist for Artificial Intelligence in Medical Imaging (CLAIM 2024), and the completed checklist is provided in the Supplementary Material.

### Patient grouping criteria

According to the 2012 revised Atlanta classification, patients were categorized as mild acute pancreatitis (MAP), MSAP, or SAP [18]. Detailed definitions are provided in Appendix S1. AP severity was determined retrospectively after completion of the index hospitalization according to the maximum severity reached during the hospital course. For model development, patients were subsequently grouped into mild (MAP) and severe (MSAP/SAP) groups.

### Clinical data collection and clinical model construction

Clinical variables collected from the hospital information system included sex, age, white blood cell (WBC), neutrophil-to-lymphocyte ratio (NLR), hematocrit, platelet, C-reactive protein (CRP), D-dimer, albumin, creatinine, triglycerides, calcium, procalcitonin (PCT), diabetes, and AP etiology. Variables associated with the severe group in univariable analysis were entered into multivariable logistic regression to identify independent predictors for clinical model construction.

### CT examination and image preprocessing

Non-contrast CT (NCCT) images were used for all pancreatic and body composition analyses. Detailed CT acquisition protocols and preprocessing parameters are provided in Appendix S1. The pancreatic parenchyma was manually delineated using ITK-SNAP (version 3.8) by Radiologist A (4 years of abdominal imaging experience). For body composition analysis, the axial CT slice at the third lumbar vertebral level (L3-level) was selected, and VAT and SM were automatically segmented using the open-source TSPC.PyQt5 tool developed in our previous work, followed by manual correction [19, 20]. The software is publicly available at https://github.com/czifan/TSPC.PyQt5.

For reproducibility assessment, 30 cases were randomly selected one month after initial segmentation. Radiologist A and Radiologist B (6 years of abdominal imaging experience) independently performed segmentation refinement and pancreatic delineation, blinded to each other’s results and clinical outcomes. Reproducibility was assessed using the Dice similarity coefficient (DSC), the 95th percentile Hausdorff distance (HD95), and the average surface distance (ASD).

### 2D and 3D DL model development and feature extraction

**Fig. 2:**
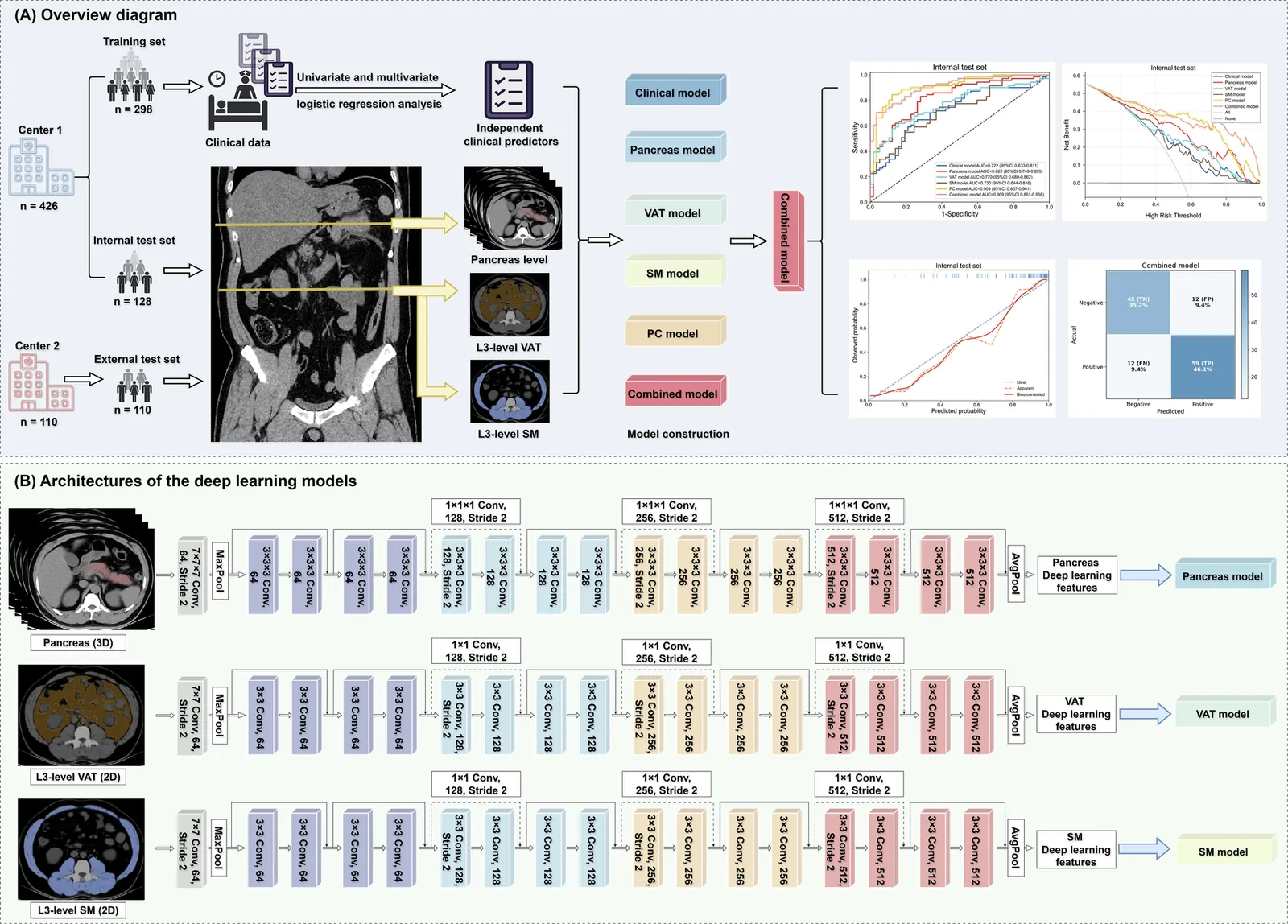
Overview of the model development workflow and DL architectures based on non-contrast CT images. **A** Overview of patient cohorts, image and clinical data processing, model construction, and performance evaluation. **B** Architectures of the 3D pancreatic DL model and the 2D VAT and SM DL models

The overall workflow is shown in Fig. 2, with detailed implementation parameters and the rationale for ResNet18 selection provided in Appendix S1. For body composition analysis, axial NCCT images at the L3 level were used to construct VAT and SM models. Segmented regions of interest (ROIs) were resized to 224 × 224 pixels, and a pretrained ResNet18 architecture was fine-tuned on the training set. Two-dimensional DL features were extracted from the penultimate average pooling layer. For pancreatic modeling, manually segmented pancreatic ROIs derived from NCCT images were cropped and resized to 128 × 128 × 32 voxels. A pretrained 3D ResNet18 architecture was fine-tuned to extract volumetric DL features.

### Model construction

Six models were constructed: clinical, pancreas, VAT, SM, pancreas-clinical (PC), and combined. The PC model integrated pancreatic DL features with selected clinical predictors, while the combined model incorporated pancreatic DL features, body composition DL features, and clinical predictors. For DL-based models, input features were standardized using *z*-score normalization, followed by feature selection using least absolute shrinkage and selection operator (LASSO). Interpretability analysis was performed using gradient-weighted class activation mapping (Grad-CAM) and SHapley Additive exPlanations (SHAP) [21, 22].

### Model comparison

The clinical, pancreas, VAT, SM, PC, and combined models were compared. Discriminative performance was assessed using receiver operating characteristic (ROC) curve analysis and the area under the curve (AUC), with differences compared using the DeLong test. Accuracy, sensitivity, specificity, positive predictive value, negative predictive value, and F1 score were also calculated. To benchmark the proposed model against an established clinical scoring system, the BISAP was additionally calculated and compared with the combined model in the training, internal test, and external test sets. Details of BISAP calculation are provided in Appendix S1. Calibration curves evaluated agreement between predicted and observed probabilities, and decision curve analysis (DCA) assessed clinical utility. Net reclassification improvement (NRI) and integrated discrimination improvement (IDI) were calculated to assess the incremental value of body composition features.

### Statistical analysis

Statistical analyses were performed using R (version 4.3.0). Normally distributed continuous variables were expressed as mean ± standard deviation and compared among the three datasets using one-way analysis of variance. Non-normally distributed variables were expressed as median with interquartile range and compared using the Kruskal–Wallis test. Categorical variables were compared using the chi-square test or Fisher exact test.

## Results

### Baseline characteristics

A total of 709 patients with AP were screened, of whom 173 were excluded because of trauma- or surgery-related AP (40 patients), neoplastic lesions (85 patients), incomplete clinical data (34 patients), and incomplete or poor-quality CT images (14 patients) (Fig. 1). The final study population comprised 536 patients (mean age, 50 years ± 16 [SD]; 360 male and 176 female), including 426 patients in the primary dataset and 110 patients in the external test set. The primary dataset was randomly divided into a training set (*n* = 298) and an internal test set (*n* = 128). Patients in the severe group (MSAP and SAP) were distributed as follows: training set, 168 of 298 (56.4%); internal test set, 71 of 128 (55.5%); and external test set, 69 of 110 (62.7%) (Table 1). Differences among the three datasets were observed for age (*p* = 0.035), platelet count (*p* = 0.018), CRP (*p* < 0.001), D-dimer (*p* < 0.001), triglycerides (*p* < 0.001), PCT (*p* < 0.001), and etiology (*p* < 0.001), whereas the other baseline variables showed no significant differences (all *p* > 0.05) (Table 1). Segmentation reproducibility results are summarized in Table S1.

**Table 1:** Clinical variables of patients with acute pancreatitis in the training, internal test, and external test sets

Variables	Training set (*n* = 298)	Internal test set (*n* = 128)	External test set (*n* = 110)	*p*-value
Sex *n* (%)				0.068
Female	106 (35.57)	44 (34.38)	26 (23.64)	
Male	192 (64.43)	84 (65.62)	84 (76.36)	
Age (years)	51.08 ± 16.21	49.27 ± 16.10	46.71 ± 15.99	0.035
WBC (10^9^/L)	12.25 ± 4.68	12.82 ± 4.49	12.11 ± 4.37	0.339
NLR	9.36 ± 7.51	10.13 ± 9.00	19.70 ± 71.75	0.81
Hematocrit (%)	44.21 ± 9.32	45.82 ± 13.93	43.53 ± 5.96	0.181
Platelet (10^9^/L)	218.71 ± 72.51	198.62 ± 62.92	217.24 ± 63.39	0.018
CRP (mg/L)	122.84 ± 116.79	122.22 ± 99.64	93.69 ± 114.10	< 0.001
D-dimer (μg/L)	2.37 ± 3.08	2.29 ± 2.74	0.80 ± 1.23	< 0.001
Albumin (g/L)	39.03 ± 6.93	39.33 ± 7.15	39.40 ± 6.13	0.378
Creatinine (μmol/L)	76.79 ± 55.53	74.16 ± 62.25	64.89 ± 30.20	0.336
Triglycerides (mmol/L)	6.19 ± 8.99	6.37 ± 7.95	13.52 ± 17.65	< 0.001
Calcium (mmol/L)	2.17 ± 0.25	2.19 ± 0.22	2.24 ± 0.18	0.085
PCT (ng/L)	2.49 ± 9.67	1.24 ± 3.92	0.41 ± 1.18	< 0.001
Diabetes				0.123
0	216 (72.48)	104 (81.25)	86 (78.18)	
1	82 (27.52)	24 (18.75)	24 (21.82)	
Etiology, *n* (%)				< 0.001
Biliary	78 (26.17)	17 (13.28)	12 (10.91)	
Hyperlipidemic	118 (39.60)	59 (46.09)	44 (40.00)	
Alcoholic	22 (7.38)	8 (6.25)	23 (20.91)	
Other	80 (26.85)	44 (34.38)	31 (28.18)	

*CRP* C-reactive protein, *NLR* neutrophil-to-lymphocyte ratio, *PCT* procalcitonin, *WBC* white blood cell count

### Comparison of the performance of predictive models

After multivariable logistic regression analysis, CRP (95% CI: 1.002–1.008, *p* = 0.004) and triglycerides (95% CI: 1.001–1.082, *p* = 0.047) were independently associated with the severe group (Table 2). The clinical model achieved AUCs of 0.729 (95% CI: 0.672–0.787), 0.722 (95% CI: 0.633–0.811), and 0.712 (95% CI: 0.604–0.819) in the training, internal test, and external test sets, respectively.

**Table 2:** Univariable and multivariable logistic regression analyses for predicting the severe group in the training set

Variables	Univariate analysis			Multivariate analysis		
	OR	95% CI	*p*-value	OR	95% CI	*p*-value
Sex	1.178	0.731–1.899	0.501			
Age	0.977	0.963–0.992	**0.002**	0.988	0.970–1.006	0.183
White blood cell	1.105	1.047–1.167	**< 0.001**	1.062	0.997–1.131	0.062
NLR	1.048	1.012–1.085	**0.008**	1.021	0.982–1.060	0.295
Hematocrit	1.021	0.994–1.049	0.131			
Platelet	1.001	0.998–1.004	0.645			
CRP	1.008	1.005–1.011	**< 0.001**	1.005	1.002–1.008	**0.004**
D-dimer	1.222	1.085–1.377	**< 0.001**	1.127	0.983–1.293	0.087
Albumin	0.951	0.918–0.986	**0.006**	0.981	0.933–1.032	0.460
Creatinine	1.004	0.999–1.008	0.158			
Triglycerides	1.066	1.031–1.103	**< 0.001**	1.040	1.001–1.082	**0.047**
Calcium	0.175	0.061–0.502	**0.001**	0.566	0.136–2.359	0.435
PCT	1.015	0.986–1.044	0.313			
Diabetes	1.605	0.949–2.715	0.078			
Etiology	1.025	0.838–1.255	0.807			

Bold font indicates a significant difference

*CI* confidence interval, *CRP* C-reactive protein, *MSAP* moderately severe acute pancreatitis, *NLR* neutrophil-to-lymphocyte ratio, *OR* odds ratio, *PCT* procalcitonin, *SAP* severe acute pancreatitis, *WBC* white blood cell count

Table 3 summarizes the performance of all models. Among the single-source models, the pancreas model showed higher AUCs than the clinical, VAT, and SM models. The PC model showed higher AUCs than the pancreas and clinical models. The combined model achieved the highest AUCs in the training and external test sets, with values of 0.897 (95% CI: 0.862–0.932) and 0.910 (95% CI: 0.856–0.964), respectively. In the internal test set, the combined model and PC model achieved identical AUCs of 0.909.

**Table 3:** Diagnostic performance of different models in the training, internal test, and external test sets

Dataset	Model	AUC (95% CI)	ACC	SEN	SPE	PPV	NPV	F1
Training set	Clinical model	0.729 (0.672–0.787)	0.708	70.2%	71.5%	76.1%	65.0%	0.731
	Pancreas model	0.833 (0.788–0.878)	0.772	70.2%	86.2%	86.8%	69.1%	0.776
	VAT model	0.789 (0.738–0.841)	0.742	79.2%	67.7%	76.0%	71.5%	0.776
	SM model	0.746 (0.692–0.801)	0.685	66.1%	71.5%	75.0%	62.0%	0.703
	PC model	0.856 (0.814–0.898)	0.802	76.8%	84.6%	86.6%	73.8%	0.814
	Combined model	0.897 (0.862–0.932)	0.826	76.2%	90.8%	91.4%	74.7%	0.831
Internal test set	Clinical model	0.722 (0.633–0.811)	0.688	63.4%	75.4%	76.3%	62.3%	0.692
	Pancreas model	0.822 (0.749–0.895)	0.781	83.1%	71.9%	78.7%	77.4%	0.808
	VAT model	0.770 (0.689–0.862)	0.719	62.0%	84.2%	83.0%	64.0%	0.710
	SM model	0.730 (0.644–0.816)	0.688	73.2%	63.2%	71.2%	65.5%	0.722
	PC model	0.909 (0.857–0.961)	0.859	84.5%	87.7%	89.6%	82.0%	0.870
	Combined model	0.909 (0.861–0.958)	0.820	70.4%	96.5%	96.2%	72.4%	0.813
External test set	Clinical model	0.712 (0.604–0.819)	0.755	81.2%	65.9%	80.0%	67.5%	0.806
	Pancreas model	0.815 (0.735–0.896)	0.755	78.3%	70.7%	81.8%	65.9%	0.800
	VAT model	0.754 (0.665–0.843)	0.691	56.5%	90.2%	90.7%	55.2%	0.696
	SM model	0.702 (0.606–0.798)	0.655	49.3%	92.7%	91.9%	52.1%	0.642
	PC model	0.851 (0.781–0.921)	0.800	88.5%	65.9%	81.3%	77.1%	0.847
	Combined model	0.910 (0.856–0.964)	0.855	88.4%	80.5%	88.4%	80.5%	0.884

*ACC* accuracy, *AUC* area under the curve, *CI* confidence interval, *NPV* negative predictive value, *PC* model pancreas-clinical model, *PPV* positive predictive value, *SEN* sensitivity, *SM* skeletal muscle, *SPE* specificity, *VAT* visceral adipose tissue

### Predictive ability of the combined model

**Fig. 3:**
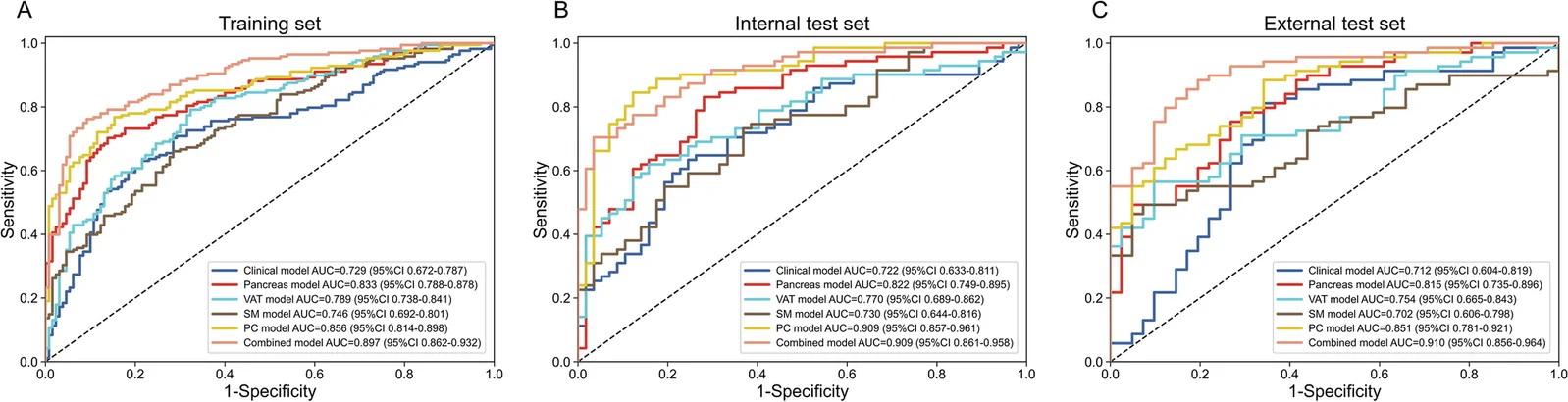
ROC curves of the six models for severity prediction in the training set (**A**), internal test set (**B**), and external test set (**C**).

The ROC curves of the combined model are shown in Fig. 3, and detailed performance metrics, including accuracy, sensitivity, specificity, PPV, and NPV, are summarized in Table 3. DeLong comparisons showed differences between the combined model and the PC model in the training set (AUC, 0.897 vs 0.856; *p* = 0.002) and external test set (AUC, 0.910 vs 0.851; *p* = 0.020), but not in the internal test set (AUC, 0.909 vs 0.909; *p* = 1.000) (Tables S2–S4).

**Fig. 4:**
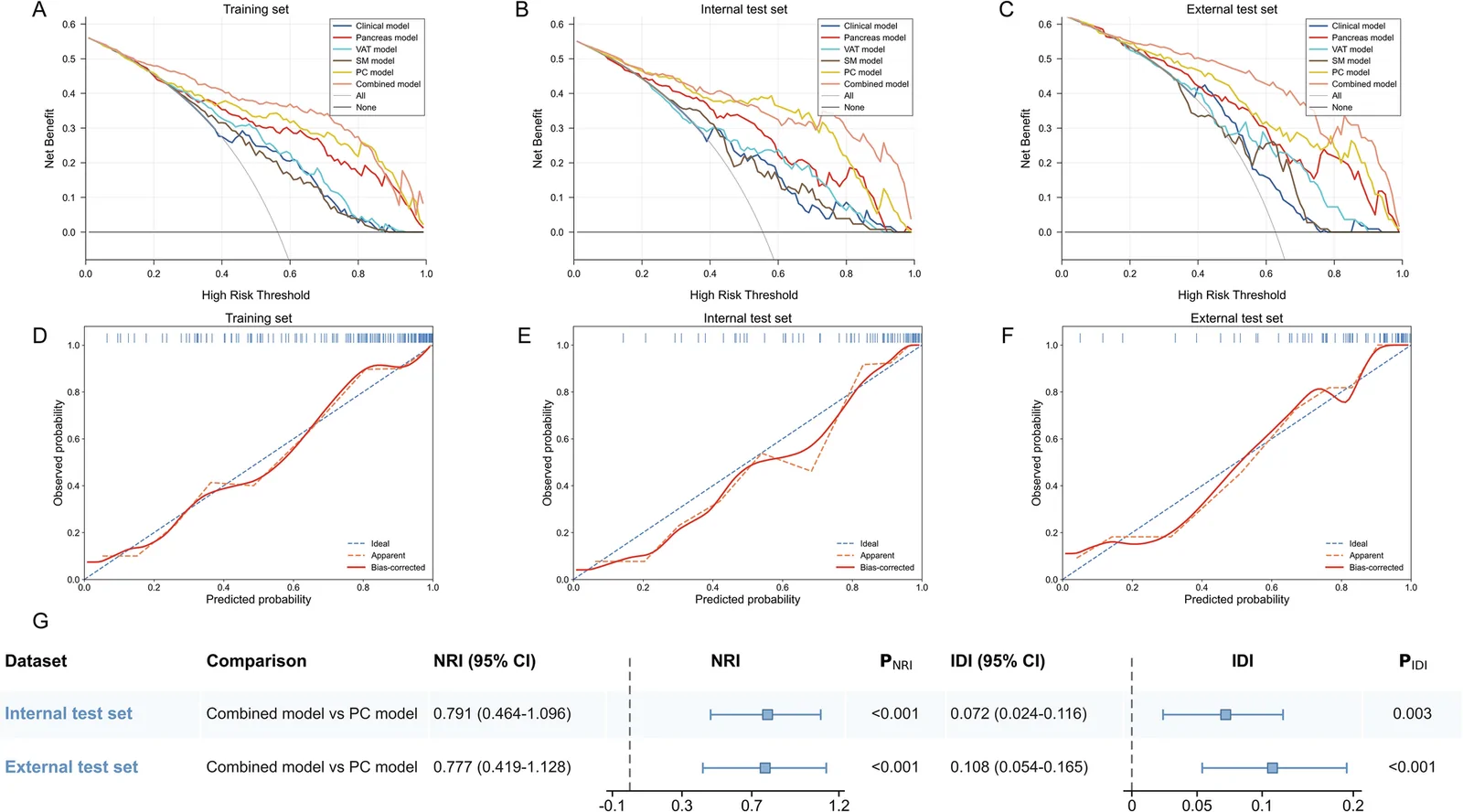
DCA (**A**–**C**), calibration curves (**D**–**F**), and NRI/IDI (**G**) analysis of the models

DCA showed higher net benefit for the combined model than the treat-all and treat-none strategies across most threshold probabilities in all datasets (Fig. 4A–C). Calibration curves showed agreement between predicted and observed probabilities in the training, internal test, and external test sets (Fig. 4D–F).

To further assess the incremental value of body composition features, NRI and IDI analyses were performed (Fig. 4G). In the internal test set, NRI was 0.791 (95% CI: 0.464–1.096; *p* < 0.001), and IDI was 0.072 (95% CI: 0.024–0.116; *p* = 0.003). In the external test set, NRI was 0.777 (95% CI: 0.419–1.128; *p* < 0.001), and IDI was 0.108 (95% CI: 0.054–0.165; *p* < 0.001). The combined model also achieved higher AUCs than BISAP in the training set (0.897 vs 0.749), internal test set (0.909 vs 0.745), and external test set (0.910 vs 0.772), with significant differences confirmed by DeLong testing (training set, *p* < 0.001; internal test set, *p* < 0.001; external test set, *p* = 0.006). Detailed diagnostic performance metrics are provided in Table S5. Confusion matrices of the combined model in the training, internal test, and external test sets are provided in Table S6.

Subgroup analysis results are shown in Fig. S1. Model-derived predicted probabilities generally increased from MAP to MSAP and SAP across models, with detailed pairwise comparisons shown in Fig. S2. For distinguishing SAP from non-SAP cases, the combined model achieved an AUC of 0.830 (Fig. S3).

### Model interpretability

**Fig. 5:**
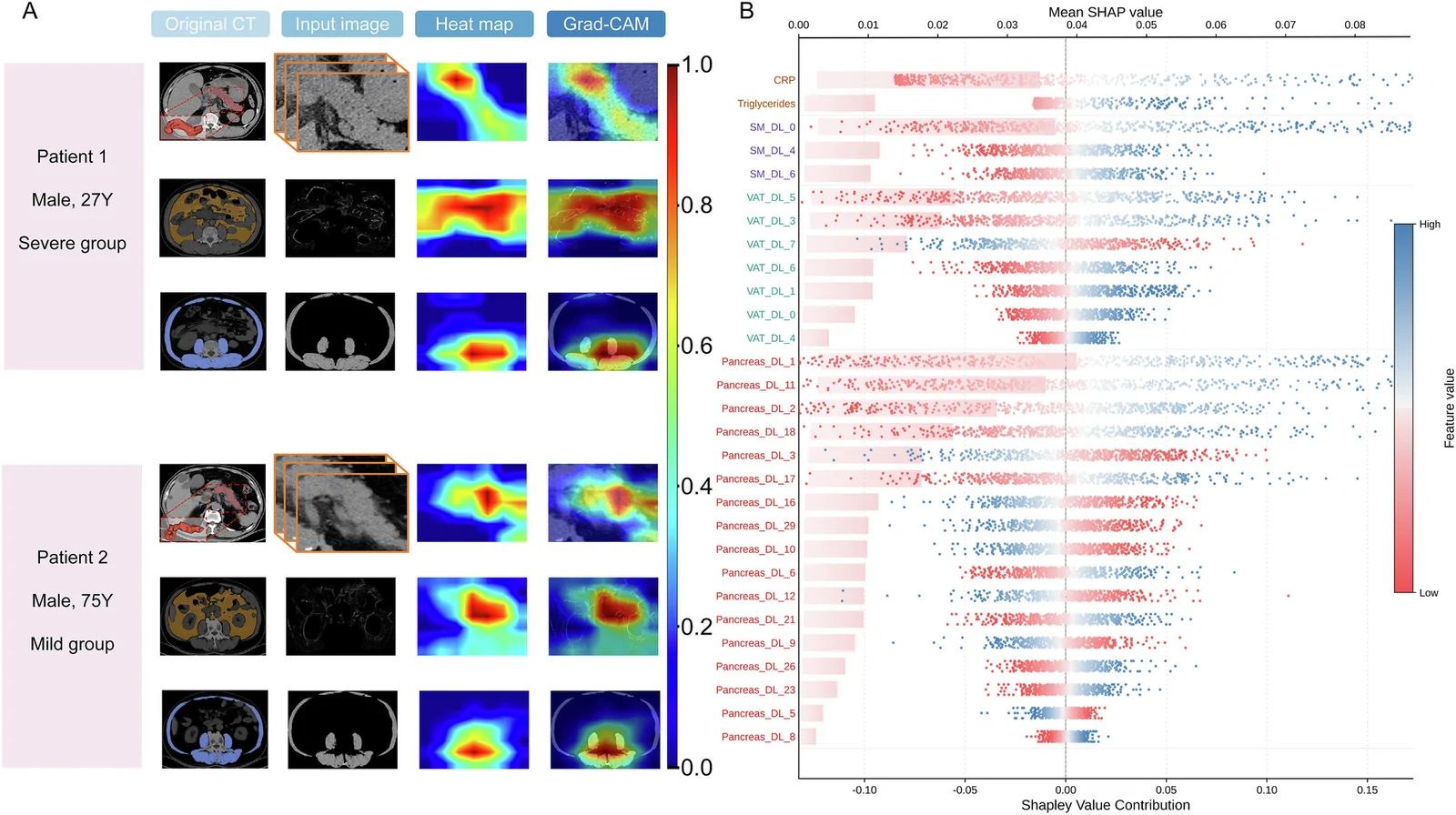
Interpretation and visualization of model predictions. **A** Grad-CAM maps for representative cases from the mild (MAP) and severe (MSAP/SAP) groups, showing model attention in the pancreas, VAT, and SM regions. Red regions indicate areas of close attention, while blue regions represent areas of lower attention. **B** SHAP summary plot of features in the combined model

Grad-CAM and SHAP results are shown in Fig. 5. Grad-CAM demonstrated distinct activation patterns between mild and severe cases, with broader activation within the pancreatic and VAT regions in the severe case compared with the mild case. SHAP demonstrated contributions from clinical, SM, VAT, and pancreatic features to model prediction.

## Discussion

In this study, a CT-based DL model integrating pancreatic imaging, CT-derived body composition, and clinical variables was developed and validated for severity prediction in AP. The combined model achieved the best performance across all datasets, demonstrating the complementary value of multi-source information. Notably, the model-derived predicted probabilities of the combined model exhibited a stepwise increase from MAP to MSAP and SAP, indicating that the model output was associated with the clinical severity gradient. Importantly, this approach leverages routinely acquired CT data without requiring additional examinations, supporting its potential for clinical implementation. Compared with BISAP, the combined model also showed consistently higher AUCs across all three datasets, further supporting the additional discriminatory value of CT-derived multi-source imaging features beyond conventional early clinical scoring. A comparison with recently published CT-based or CT-containing DL studies for AP severity prediction is provided in Supplementary Table S7. The main contribution of the present study is the integration of pancreatic imaging, CT-derived body composition, and clinical variables within a multi-source DL framework.

Body composition measured at the L3 level on CT is widely accepted as a surrogate marker of whole-body muscle and adipose tissue distribution because of its standardized anatomical location and good reproducibility [23]. In the present study, the satisfactory inter- and intra-observer agreement further supported the robustness of CT-based body composition assessment. Body composition has also been increasingly recognized as an important factor influencing the clinical course of AP. VAT may aggravate pancreatic injury through the release of pro-inflammatory mediators and free fatty acids, whereas SM reflects metabolic reserve and tolerance to systemic inflammatory stress. A recent systematic review by Tan et al [24] demonstrated that CT-quantified VAT was associated with increased severity and complications of AP, while Xie et al [25] reported that increased VAT and a higher visceral fat-to-muscle ratio were associated with more severe disease. Using alternative body composition metrics, Horibe et al [26] showed that a CT-derived body composition *z*-score was associated with AP severity, and Kirzhner et al [27] demonstrated that reduced psoas muscle index and density were associated with worse clinical outcomes. However, these studies primarily relied on predefined imaging parameters or simple area-based body composition metrics, which may not fully capture complex imaging characteristics associated with disease severity. Compared with conventional approaches, DL enables the extraction of high-dimensional imaging features from CT images and may capture subtle imaging patterns beyond visual assessment. It should be noted that VAT-derived DL features in AP may not represent only pre-existing visceral adiposity. In this study, VAT was analyzed using segmented L3-level VAT ROIs rather than whole abdominal CT images, which reduced the direct influence of peripancreatic fluid collections and other non-VAT structures. However, AP-related inflammatory changes may still affect imaging patterns within the VAT region and contribute to model prediction. Therefore, the VAT model should be interpreted as capturing a composite imaging signal reflecting visceral fat burden, imaging patterns within the VAT region, and AP-related inflammatory changes, rather than as a pure measure of VAT amount alone.

Because AP severity is influenced by both local pancreatic injury and systemic inflammatory-metabolic responses, integrating pancreatic imaging with body composition may provide a more comprehensive assessment of disease severity [7]. In the present study, DL models were separately developed using pancreatic regions and L3-level body composition images, enabling simultaneous characterization of local pancreatic injury and systemic host status. Consistent with this concept, the pancreas model showed higher discriminative performance than the clinical and single body composition models, underscoring the importance of directly characterizing pancreatic injury. Furthermore, the combined model demonstrated superior performance over both single-source models and the PC model, suggesting that body composition provides complementary predictive information beyond pancreatic imaging and clinical biomarkers alone. These findings support the potential value of multi-source DL-based CT analysis for imaging-based risk stratification in AP.

Among the clinical variables, CRP and triglycerides were identified as important predictors in the present study. CRP is an acute-phase protein synthesized by the liver in response to inflammatory cytokines such as interleukin-6 and reflects the magnitude of systemic inflammatory response. In this study, higher CRP levels were independently associated with increased risk of belonging to the severe group, which is consistent with previous studies reporting the association between inflammatory biomarkers and disease severity [28, 29]. Triglycerides were also identified as an important factor, as hypertriglyceridemia is one of the recognized etiologies of AP and may aggravate pancreatic injury through the generation of toxic free fatty acids and amplification of inflammatory responses [30, 31]. The inclusion of these laboratory indicators together with imaging features may therefore provide complementary information by reflecting both systemic inflammatory status and metabolic risk factors. These findings further support the value of integrating clinical biomarkers with multi-source imaging features for a more comprehensive assessment of disease severity in AP.

Grad-CAM and SHAP provided complementary interpretability information. Grad-CAM offered spatial-level explanations by highlighting image regions that contributed to model prediction. The broader activation within pancreatic and VAT regions in severe cases was consistent with the superior performance of the pancreas model and the added value of VAT-derived information. In contrast, SHAP provided feature-level explanations by estimating the relative contribution of clinical and DL-derived features. The importance of CRP and triglycerides in SHAP analysis was consistent with the multivariable clinical analysis and supported the integration of inflammatory and metabolic biomarkers with imaging features. Clinically, Grad-CAM may help assess whether model attention is anatomically plausible, whereas SHAP may help identify the features driving individualized risk prediction. Together, these methods improve the transparency and clinical acceptability of the proposed DL model.

This study has several limitations. First, this retrospective design may introduce selection bias despite the inclusion of consecutive patients from two centers. Second, although external testing was performed, all patients were recruited from institutions within the same country, which may limit generalizability to other populations and healthcare settings. CT acquisition protocols also differed between centers, including scanner vendors and reconstruction thickness. Although all CT images were resampled to a uniform voxel spacing, resampling cannot recover information lost because of thicker-slice reconstruction or fully eliminate vendor-related image differences, which may have affected the generalizability of the 3D pancreatic model in external testing. Third, the limited sample size may reduce the stability of some estimates. The slightly lower training AUC than the internal and external test AUCs may be related to sampling variability, differences in case mix, and the effects of regularization and feature selection. Therefore, the external test performance should be interpreted cautiously and confirmed in larger prospective multicenter cohorts. Fourth, body composition analysis was limited to VAT and SM at the L3-level rather than whole-abdominal assessment, which may not fully capture regional heterogeneity. Fifth, pancreatic segmentation was manually performed and may introduce observer dependency despite satisfactory reproducibility. Automated segmentation may improve reproducibility and facilitate clinical implementation. Finally, although Grad-CAM and SHAP improved interpretability, the internal decision-making process of the DL model remains only partially explainable.

## Conclusion

In conclusion, this study developed and validated a DL-based CT model integrating pancreatic imaging, body composition, and clinical variables for severity prediction in AP. The addition of body composition information improved predictive performance beyond pancreatic imaging and clinical features alone. These findings support the potential value of multi-source CT-based analysis for early AP risk stratification. Future prospective multicenter studies with larger and more diverse populations, together with fully automated segmentation workflows, are warranted to further validate and improve this approach.

## Supplementary information

ELECTRONIC SUPPLEMENTARY MATERIAL

## Data Availability

The datasets used and/or analyzed during the current study are available from the corresponding author on reasonable request.

## Abbreviations

AP: Acute pancreatitis
AUC: Area under the curve
BISAP: Bedside index for severity in acute pancreatitis
CRP: C-reactive protein
CT: Computed tomography
CTSI: CT severity index
DCA: Decision curve analysis
DL: Deep learning
Grad-CAM: Gradient-weighted class activation mapping
IDI: Integrated discrimination improvement
MAP: Mild acute pancreatitis
MSAP: Moderately severe acute pancreatitis
NLR: Neutrophil-to-lymphocyte ratio
NRI: Net reclassification improvement
PC: Pancreas-clinical
PCT: Procalcitonin
ROC: Receiver operating characteristic
ROI: Region of interest
SAP: Severe acute pancreatitis
SHAP: Shapley additive explanations
SM: Skeletal muscle
VAT: Visceral adipose tissue
WBC: White blood cell count
