# Supplementary material for: CT-based deep learning integrating pancreatic imaging and body composition for severity prediction in acute pancreatitis
Source: Insights Imaging. 2026 Oct 28;17:266. doi: 10.1186/s13244-026-02443-w (PMC13719943; doi:10.1186/s13244-026-02443-w)
Supplement: Supplementary file 1 [file 13244_2026_2443_MOESM1_ESM.pdf]

CT-based deep learning integrating pancreatic imaging and body composition for severity prediction in acute pancreatitis

ELECTRONIC SUPPLEMENTARY MATERIAL

Appendix S1

Materials and methods

AP diagnostic criteria

Acute pancreatitis (AP) was diagnosed when at least two of the following three criteria were met: (a) persistent upper abdominal pain; (b) serum amylase and/or lipase levels more than three times the upper limit of normal; and (c) imaging findings consistent with AP.

Data handling

All imaging and clinical data were de-identified before analysis. No data imputation was performed. Patients with incomplete clinical data required for analysis or incomplete/poor-quality CT images were excluded.

Patient grouping criteria

According to the 2012 revised Atlanta classification, AP was categorized as mild acute pancreatitis (MAP), moderately severe acute pancreatitis (MSAP), and severe acute pancreatitis (SAP). MAP was defined as AP without organ failure or local/systemic complications. MSAP was defined as transient organ failure lasting less than 48 hours and/or local or systemic complications without persistent organ failure. SAP was defined as persistent organ failure lasting 48 hours or longer, involving one or more organ systems. Severity classification was used as the reference standard and was determined according to the 2012 revised Atlanta classification based on clinical records, laboratory data, imaging findings, and organ failure documentation.

Data partitioning

The primary dataset from Center 1 was randomly divided into the training set and internal test set at a ratio of 7:3. Data partitioning was performed at the patient level, with no patient overlap among the training, internal test, and external test sets. The external test set consisted of all eligible patients from Center 2.

CT examination and image preprocessing

Abdominal non-contrast CT (NCCT) images were used as model inputs for all enrolled patients. The examinations were performed at the two participating centers using multidetector CT scanners. All scans were performed in the supine position with feet first, covering the region from the diaphragmatic dome to the inferior border of the pubic symphysis to allow simultaneous evaluation of pancreatic parenchyma as well as L3-level skeletal muscle and visceral adipose tissue.

At Xuanwu Hospital Capital Medical University (Center 1), CT scans were performed using either a GE Revolution CT scanner (GE Healthcare, USA) or a Siemens Somatom

Force scanner (Siemens Healthineers, Germany). The scanning parameters were as follows: tube voltage of 120 kVp, automatic tube current modulation with a maximum tube current of 200 mAs, detector collimation of 64 × 0.625 mm, pitch of 0.984, rotation time of 0.5 s, and a matrix size of 512 × 512. The slice thickness and reconstruction interval were both set to 5 mm.

At Chongqing General Hospital (Center 2), CT scans were performed using a Philips IQon Spectral CT scanner (Philips Healthcare, Netherlands). The scanning parameters were as follows: tube voltage of 120 kVp, automatic tube current modulation, detector collimation of 64 × 0.625 mm, pitch of 1.2, rotation time of 0.5 s, and a matrix size of 512 × 512. The slice thickness and reconstruction interval were both set to 1 mm.

Prior to segmentation, all CT images were resampled to an isotropic voxel spacing of 1 × 1 × 1 mm^3, and image intensities were standardized using a fixed window setting (window width = 300 HU, window level = 35 HU). No explicit center harmonization or domain adaptation was performed. Image preprocessing, including isotropic resampling, fixed window setting, and intensity normalization, was used to reduce protocol-related variability.

2D and 3D DL model development and feature extraction

Each patient contributed one L3-level axial NCCT image for VAT modeling, one L3-level axial NCCT image for SM modeling, and one 3D pancreatic ROI derived from NCCT images for pancreatic modeling. Thus, the numbers of original 2D VAT images and original 2D SM images were 298, 128, and 110 in the training, internal test, and external test sets, respectively. For the pancreatic model, the corresponding numbers of 3D pancreatic ROI inputs were 298, 128, and 110, and each ROI was resized to 128 × 128 × 32 voxels before model input.

Deep learning model development and feature extraction were performed using the built-in ResNet18 architecture in the OnekeyAI platform (version 6.3.15). For body composition analysis, axial NCCT images at the L3-level were used to construct VAT and SM models. Segmented ROIs were resized to a fixed dimension of 224 × 224 pixels using bilinear interpolation. Data augmentation strategies, including random flipping and random cropping, were applied only during training and did not change the number of independent patient-level samples. A ResNet18 architecture pretrained on the ImageNet dataset was adopted as the backbone network, and transfer learning was performed by fine-tuning pretrained weights on the training set. Images were normalized using ImageNet normalization parameters to ensure consistency with the input distribution of the pretrained network. The 2D models were trained using the stochastic gradient descent optimizer with an initial learning rate of 0.01, a batch size of 32, and 50 epochs. After training, 2D DL features were extracted from the penultimate average pooling layer of the ResNet18 network for subsequent analysis.

For pancreatic modeling, manually segmented pancreatic ROIs were cropped and resized to 128 × 128 × 32 voxels. All NCCT images were resampled to isotropic voxel spacing prior to model input. A 3D ResNet18 architecture was used to extract volumetric imaging features, followed by training on the training set. The 3D model was trained using the Adam optimizer with an initial learning rate of 0.0001, a batch size of 8, 120 epochs, and a weight decay of 0.0001. After training, 3D DL features were extracted from the

penultimate average pooling layer of the 3D ResNet18 network.

No separate tuning subset was used. Training was performed using predefined hyperparameters, and the internal and external test sets were used only for model evaluation.

DL features derived from the pancreas, VAT, and SM models were subsequently standardized using z-score normalization. Feature selection was then performed using least absolute shrinkage and selection operator regression within the training set. Selected features were used for subsequent model construction and fusion analysis.

Rationale for ResNet18 selection

ResNet18 was prespecified as the backbone architecture for the primary analysis before evaluation of the internal and external test sets. This architecture was selected because ResNet-based models have been widely used in medical image analysis and provide a practical trade-off between feature-learning capacity and model complexity in clinical imaging tasks [1]. Its residual connections facilitate model optimization and gradient propagation through skip connections, thereby supporting stable training of deep neural networks [2]. Compared with deeper convolutional neural networks or transformer-based architectures, ResNet18 has a relatively compact structure and lower computational burden, which is relevant for medical imaging datasets with a limited number of labeled cases [3]. In addition, established 2D and 3D implementations of ResNet18 enabled the use of a consistent feature-extraction framework for L3-level body composition images and volumetric pancreatic ROIs.

After the ResNet18 backbone had been prespecified for the primary models, additional architecture analyses were conducted as post hoc exploratory sensitivity analyses. The same ROI inputs, patient-level data partitions, and outcome definitions were used for each comparison. For the pancreatic model, ResNet18, DenseNet121, and Vision Transformer were evaluated, whereas ResNet18 and DenseNet121 were evaluated for the VAT and SM models. DenseNet121 and Vision Transformer showed near-perfect training performance for the pancreatic model but substantially lower performance in the internal and external test sets, while DenseNet121 did not consistently improve test-set performance for the body composition models. These findings are reported descriptively to illustrate the behavior of alternative backbone architectures. They were not used to select, tune, or modify the prespecified ResNet18-based primary models and should not be interpreted as a formal architecture-selection procedure. Detailed results of these exploratory analyses are provided in Tables S8 and S9.

BISAP calculation

The BISAP score was retrospectively calculated using five components: blood urea nitrogen level >25 mg/dL, impaired mental status, systemic inflammatory response syndrome, age >60 years, and pleural effusion.

Statistical analysis

Sample size considerations

Patients with MSAP or SAP accounted for approximately 57.5% of the study population. Using a two-sided 95% confidence interval and an allowable error of 10% of the event proportion, the estimated minimum sample size was approximately 284 patients according to the following formula:

$$n = Z^2 \times p(1 - p) / d^2$$

where $Z = 1.96$, $p = 0.575$, and $d = 0.0575$. The final study population of 536 patients exceeded this requirement.

Table S1 Reproducibility of Pancreatic and Body Composition Segmentation

Structure	Observer comparison	DSC (mean ± SD)	HD95 (mm) (mean ± SD)	ASD (mm) (mean ± SD)
Pancreas	Inter-observer	0.8627 ± 0.0667	7.1122 ± 5.4865	1.1877 ± 0.7016
	Intra-observer	0.9648 ± 0.0249	2.4612 ± 2.9168	0.3335 ± 0.2842
VAT	Inter-observer	1.0000 ± 0.0001	0.0000 ± 0.0000	0.0001 ± 0.0005
	Intra-observer	1.0000 ± 0.0001	0.0000 ± 0.0000	0.0001 ± 0.0005
SM	Inter-observer	0.9999 ± 0.0002	0.0000 ± 0.0000	0.0002 ± 0.0006
	Intra-observer	0.9999 ± 0.0002	0.0000 ± 0.0000	0.0001 ± 0.0002

DSC Dice similarity coefficient, HD95 95th percentile Hausdorff distance, ASD average surface distance, SM skeletal muscle, VAT visceral adipose tissue.

Table S2 Pairwise DeLong Test Comparisons of Model AUCs in the Training Set

**Model (AUC)**	**Clinical model**	**Pancreas model**	**VAT model**	**SM model**	**PC model**	**Combined model**
Clinical model (AUC = 0.729)	-	0.003*	0.095	0.655	<0.001*	<0.001*
Pancreas model (AUC = 0.833)	0.003*	-	0.179	0.011*	0.019*	<0.001*
VAT model (AUC = 0.789)	0.095	0.179	-	0.213	0.032*	<0.001*
SM model (AUC = 0.746)	0.655	0.011*	0.213	-	<0.001*	<0.001*
PC model (AUC = 0.856)	<0.001*	0.019*	0.032*	<0.001*	-	0.002*
Combined model (AUC = 0.897)	<0.001*	<0.001*	<0.001*	<0.001*	0.002*	-

AUC area under the curve, VAT visceral adipose tissue, SM skeletal muscle, PC model pancreas-clinical model.
*p < 0.05 indicates statistical significance.

Table S3 Pairwise DeLong Test Comparisons of Model AUCs in the Internal Test Set

**Model (AUC)**	**Clinical model**	**Pancreas model**	**VAT model**	**SM model**	**PC model**	**Combined model**
Clinical model (AUC = 0.722)	-	0.117	0.393	0.894	<0.001*	<0.001*
Pancreas model (AUC = 0.822)	0.117	-	0.894	0.075	<0.001*	0.004*
VAT model (AUC = 0.770)	0.393	0.894	-	0.416	0.004*	<0.001*
SM model (AUC = 0.730)	0.894	0.075	0.416	-	<0.001*	<0.001*
PC model (AUC = 0.909)	<0.001*	<0.001*	0.004*	<0.001*	-	1.000
Combined model (AUC = 0.909)	<0.001*	0.004*	<0.001*	<0.001*	1.000	-

AUC area under the curve, VAT visceral adipose tissue, SM skeletal muscle, PC model pancreas-clinical model.
*p < 0.05 indicates statistical significance.

Table S4 Pairwise DeLong Test Comparisons of Model AUCs in the External Test Set

Model (AUC)	Clinical model	Pancreas model	VAT model	SM model	PC model	Combined model
Clinical model (AUC = 0.712)	-	0.139	0.566	0.901	0.006*	<0.001*
Pancreas model (AUC = 0.815)	0.139	-	0.345	0.083	0.180	0.008*
VAT model (AUC = 0.754)	0.566	0.345	-	0.451	0.114	0.002*
SM model (AUC = 0.702)	0.901	0.083	0.451	-	0.022*	<0.001*
PC model (AUC = 0.851)	0.006*	0.180	0.114	0.022*	-	0.020*
Combined model (AUC = 0.910)	<0.001*	0.008*	0.002*	<0.001*	0.020*	-

AUC area under the curve, VAT visceral adipose tissue, SM skeletal muscle, PC model pancreas-clinical model.
**p* < 0.05 indicates statistical significance.

Table S5 Diagnostic performance of the BISAP and combined model in the training, internal test, and external test sets

Dataset	Model	AUC (95%CI)	*p*-value	ACC	SEN	SPE	PPV	NPV	F1
Training set	BISAP	0.749 (0.699-0.800)		0.721	0.899	0.492	0.696	0.790	0.784
	Combined model	0.897 (0.862-0.932)	< 0.001	0.826	0.762	0.908	0.914	0.747	0.831
Internal test set	BISAP	0.745 (0.668-0.822)		0.703	0.845	0.526	0.690	0.732	0.759
	Combined model	0.909 (0.861-0.958)	< 0.001	0.820	0.704	0.965	0.962	0.724	0.813
External test set	BISAP	0.772 (0.688-0.855)		0.645	0.464	0.951	0.941	0.513	0.621
	Combined model	0.910 (0.856-0.964)	0.006	0.855	0.884	0.805	0.884	0.805	0.884

ACC accuracy, AUC area under the curve, BISAP bedside index for severity in acute pancreatitis, CI confidence interval, NPV negative predictive value, PPV positive predictive value, SEN sensitivity, SPE specificity.

p-values were calculated using the DeLong test for comparison of AUCs between BISAP and the combined model.

Table S6 Confusion matrices of the combined model in the training, internal test, and external test sets

Dataset	TN	FP	FN	TP
Training set	118	12	40	128
Internal test set	55	2	21	50
External test set	33	8	8	61

TN true negative, FP false positive, FN false negative, TP true positive.

The mild group included MAP cases and was defined as the negative class, whereas the severe group included MSAP and SAP cases and was defined as the positive class.

Table S7 Comparison with recently published deep learning-based studies for acute pancreatitis severity prediction

Study	Year	Sample size	Input	Model	Target	Validation	Performance	Main difference
Chen et al. [4]	2023	978 patients; development 783, validation 195	NCCT ≤72 h ± clinical markers	CNN-based image DL; image-clinical DL	MAP/MSAP/SAP	Internal validation	Combined DL: AUC 0.820 for MAP, 0.920 for SAP; image-only: AUC 0.741 for MAP, 0.896 for SAP	No external test; no body composition
Liang et al. [5]	2023	1,798 enhanced CT scans from 1,561 patients	Portal venous CECT	3D DenseNet	CTSI and Atlanta severity labels	Internal test	Macro-AUC 0.980 for CTSI labels; 0.864 for Atlanta labels	Enhanced scan-level data; no external test; no clinical/body composition input
Yin et al. [6]	2024	1,221 development/internal; 266 external	EHR, CT radiomics, 2D CT DL	PrismSAP multimodal fusion	SAP vs non-SAP	Internal and external test	External AUC 0.916; DL-only external AUC 0.687	Multimodal fusion; no L3 VAT/SM DL features
Xu et al. [7]	2026	Fine-tuning 550; internal 100; external 518	Admission CECT ≤24 h	SSL ViT/DINO; hybrid with mCTSI/BISAP	SAP and MAP prediction	Internal and international external test	External AUC 0.887 for SAP, 0.858 for MAP; hybrid SAP AUC 0.913	Large SSL-based study; no body composition
Present study	2026	536 patients; training 298, internal 128, external 110	NCCT ≤72 h; pancreas ROI; L3 VAT/SM; clinical variables	3D/2D ResNet18 combined model	MSAP/SAP vs MAP	Internal and external test	AUC 0.897, 0.909, 0.910 in training, internal, external sets	Integrates pancreas, body composition, and clinical variables

AP acute pancreatitis, AUC area under the curve, CECT contrast-enhanced computed tomography, CNN convolutional neural network, CT computed tomography, CTSI computed tomography severity index, DL deep learning, EHR electronic health record, MAP mild acute pancreatitis, mCTSI modified

computed tomography severity index, MSAP moderately severe acute pancreatitis, NCCT noncontrast computed tomography, SAP severe acute pancreatitis, SSL self-supervised learning, SM skeletal muscle, VAT visceral adipose tissue.
Because the included studies differed in CT timing, input data, prediction target, outcome definition, and validation strategy, the reported performance metrics should be interpreted as contextual benchmarks rather than direct head-to-head comparisons.

Table S8 Exploratory architecture comparison for the pancreas model

Architecture	Dataset	AUC (95%CI)	ACC	SEN	SPE	PPV	NPV	F1
Vision Transformer	Training set	1.000（1.000-1.000）	1.000	1.000	1.000	1.000	1.000	1.000
	Internal test set	0.599（0.499-0.700）	0.641	0.690	0.579	0.671	0.600	0.681
	External test set	0.581（0.470-0.692）	0.636	0.783	0.390	0.684	0.516	0.730
DenseNet 121	Training set	0.998（0.994-1.000）	0.993	1.000	0.985	0.988	1.000	0.994
	Internal test set	0.725（0.637-0.812）	0.672	0.535	0.842	0.809	0.593	0.644
	External test set	0.789（0.704-0.873）	0.755	0.739	0.780	0.850	0.640	0.791
ResNet 18	Training set	0.833 (0.788-0.878)	0.772	0.702	0.862	0.868	0.691	0.776
	Internal test set	0.822 (0.749-0.895)	0.781	0.831	0.719	0.787	0.774	0.808
	External test set	0.815 (0.735-0.896)	0.755	0.783	0.707	0.818	0.659	0.800

ACC accuracy, AUC area under the curve, CI confidence interval, NPV negative predictive value, PPV positive predictive value, SEN sensitivity, SPE specificity.

All architectures were evaluated using the same pancreatic ROI inputs, fixed patient-level data partitions, and outcome definition. This post hoc exploratory analysis was conducted after prespecification of the ResNet18-based primary model and was not used for model selection, hyperparameter tuning, or modification of the primary model.

Table S9 Exploratory architecture comparison for the body composition models

Architecture	Model	Dataset	AUC （95%CI）	ACC	SEN	SPE	PPV	NPV	F1
DenseNet 121	VAT model	Training set	0.828 （0.782-0.874）	0.762	0.827	0.677	0.768	0.752	0.797
		Internal test set	0.728 （0.642-0.815）	0.688	0.732	0.632	0.712	0.655	0.722
		External test set	0.738 （0.640-0.837）	0.718	0.725	0.707	0.806	0.604	0.763
	SM model	Training set	0.649 （0.587-0.712）	0.631	0.738	0.492	0.653	0.593	0.693
		Internal test set	0.676 （0.582-0.770）	0.680	0.648	0.719	0.742	0.621	0.692
		External test set	0.672 （0.569-0.775）	0.636	0.594	0.707	0.774	0.509	0.672
	VAT model	Training set	0.789 (0.738-0.841)	0.742	0.792	0.677	0.760	0.715	0.776
		Internal test set	0.770 (0.689-0.862)	0.719	0.620	0.842	0.830	0.640	0.710
		External test set	0.754 (0.665-0.843)	0.691	0.565	0.902	0.907	0.552	0.696
		Training set	0.746 (0.692-0.801)	0.685	0.661	0.715	0.750	0.620	0.703
		Internal test set	0.730 (0.644-0.816)	0.688	0.732	0.632	0.712	0.655	0.722

ResNet 18	SM model	External test set	0.702 (0.606-0.798)	0.655	0.493	0.927	0.919	0.521	0.642

ACC accuracy, AUC area under the curve, CI confidence interval, NPV negative predictive value, PPV positive predictive value, SEN sensitivity, SPE

specificity.

All architectures were evaluated using the same L3-level segmented ROI inputs, fixed patient-level data partitions, and outcome definition within each body composition model. This post hoc exploratory analysis was conducted after prespecification of the ResNet18-based primary models and was not used for model selection, hyperparameter tuning, or modification of the primary models.

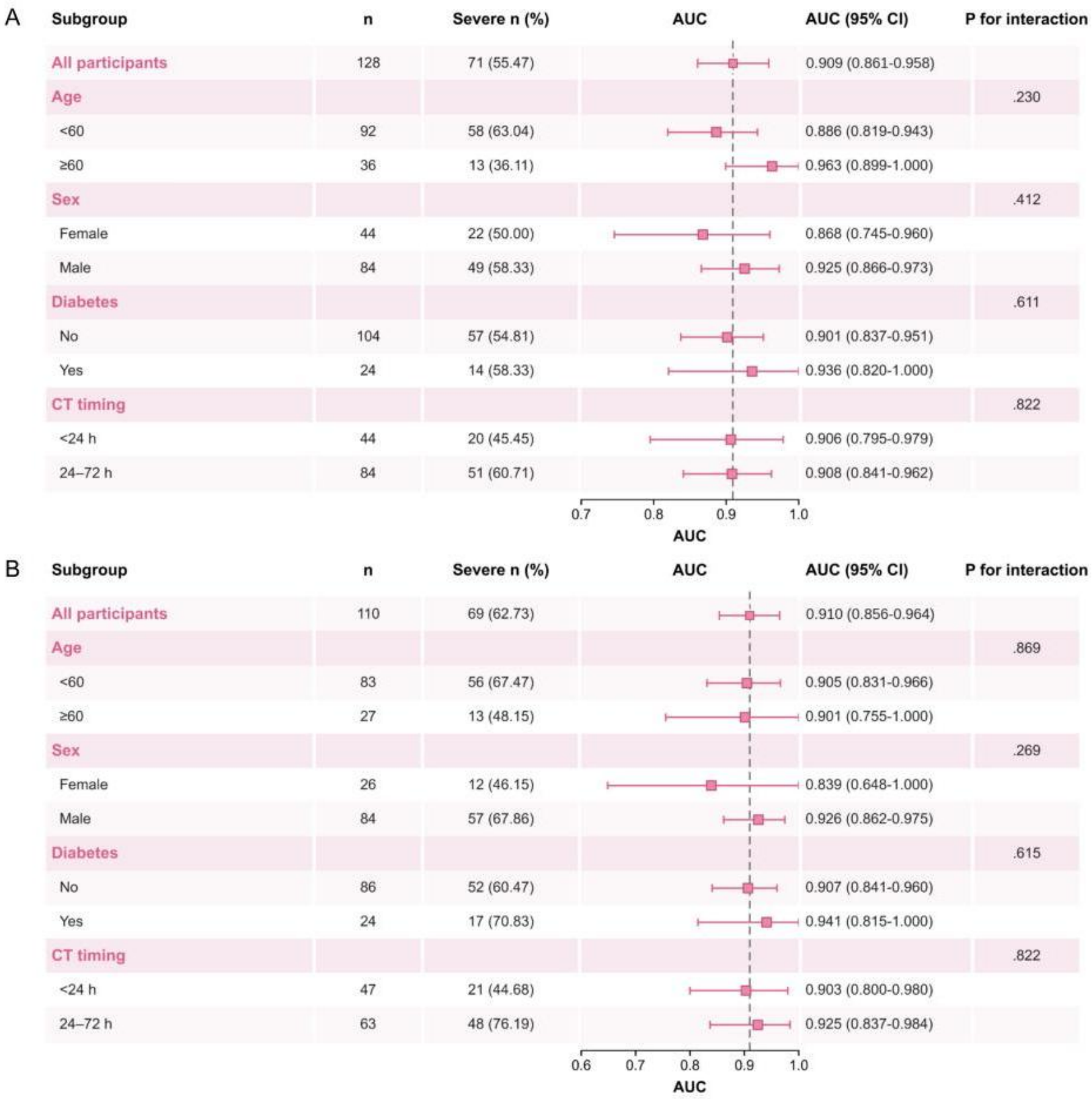

Figure S1. Subgroup analysis of the combined model in the internal test set (A) and external test set (B). Model performance was evaluated according to age, sex, diabetes status, and CT timing from symptom onset. CT timing was stratified as <24 h and 24-72 h. The combined model showed generally consistent performance across subgroups, without evidence of a significant interaction by CT timing. P values for interaction were used to assess whether model performance differed across subgroups.

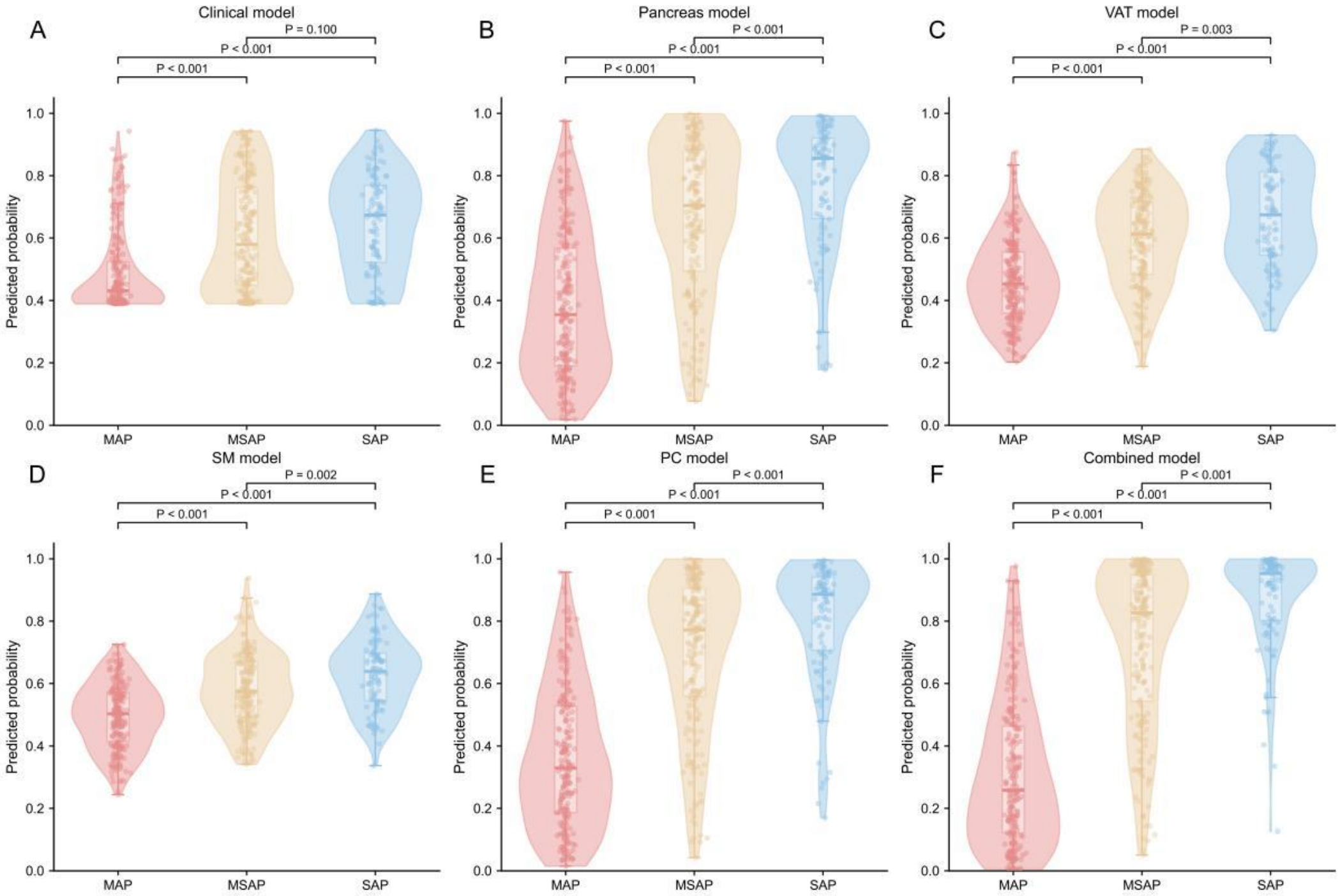

Figure S2 Distribution of predicted probabilities across MAP, MSAP, and SAP groups

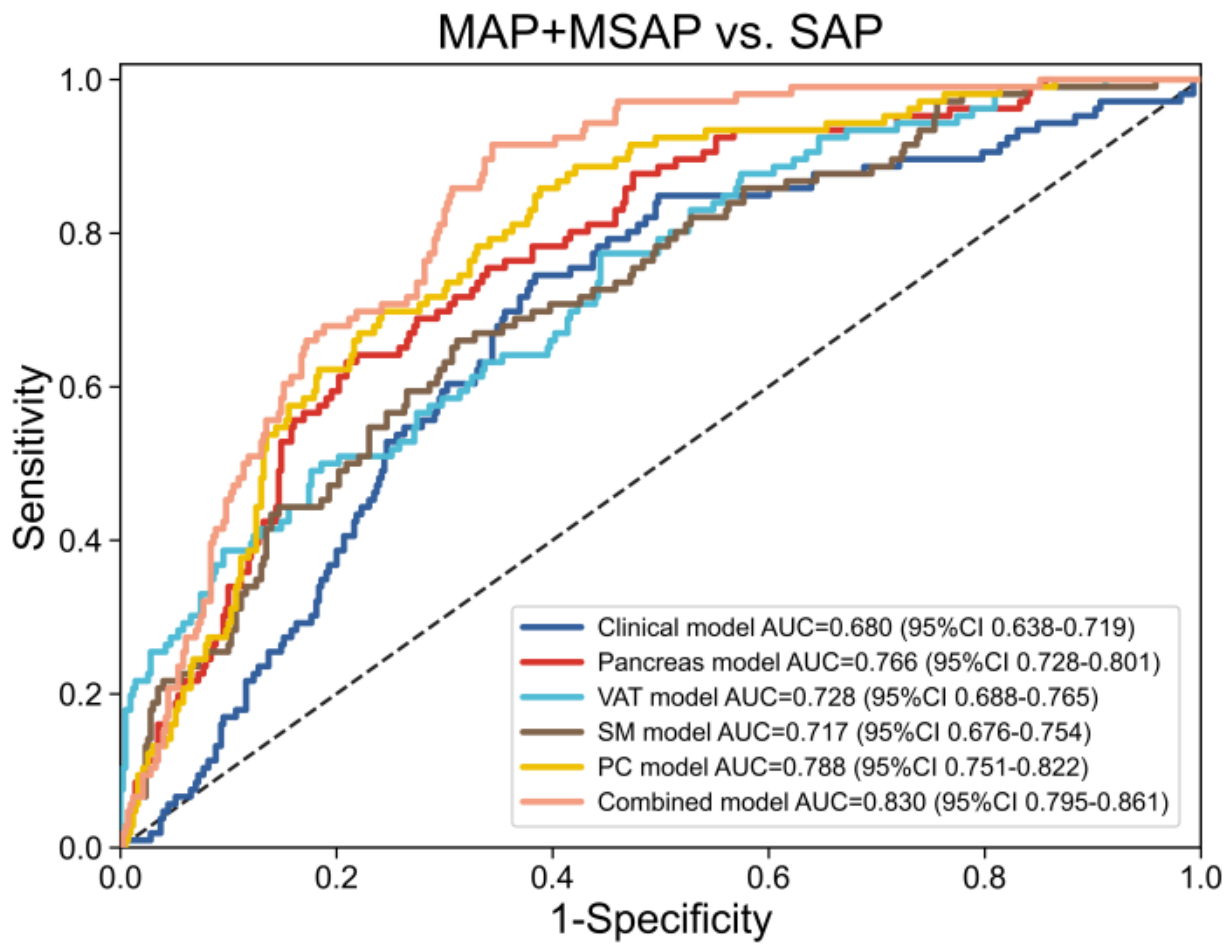

Figure S3 Receiver operating characteristic curves of the combined model for differentiating SAP from non-SAP patient

CLAIM 2024 Checklist

Section/Topic	No.	Item	Yes/No/NA	Location	Comments
TITLE/ABSTRACT	**1**	Identification as a study of AI methodology, specifying the category of technology used (eg, deep learning)	**Yes**	Main text: Title; Abstract, Objectives/Methods/Results/Conclusions	The title and abstract identify a CT-based deep learning study for AP severity prediction.
ABSTRACT	**2**	Summary of study design, methods, results, and conclusions	**Yes**	Main text: Abstract	The structured abstract reports retrospective dual-center design, patient numbers, partitions, models, statistics, AUCs, and conclusions.
INTRODUCTION	**3**	Scientific and/or clinical background, including the intended use and role of the AI approach	**Yes**	Main text: Introduction	The introduction summarizes AP severity prediction, limitations of clinical scores, CT body composition, and DL rationale.
INTRODUCTION	**4**	Study aims, objectives, and hypotheses	**Yes**	Main text: Introduction; Abstract Objectives	The study aim is to develop and validate a DL-based CT model integrating pancreatic imaging, body composition, and clinical variables.
METHODS - Study Design	**5**	Prospective or retrospective study	**Yes**	Main text: Materials and methods, Patients	Reported as a retrospective dual-center study with ethics approvals and waived consent.
METHODS - Study Design	**6**	Study goal	**Yes**	Main text: Introduction; Patient	Goal is severity prediction/risk

				grouping criteria; Conclusion	stratification, classifying MAP versus MSAP/SAP.
METHODS - Data	**7**	Data sources	**Yes**	Main text: Patients; Supplement Appendix S1, CT examination	Data were from two tertiary centers, with center-specific inclusion periods and CT scanners reported.
METHODS - Data	**8**	Inclusion and exclusion criteria	**Yes**	Main text: Patients; Results; Figure 1	Inclusion/exclusion criteria, consecutive screening, and excluded patient counts by reason are reported.
METHODS - Data	**9**	Data preprocessing	**Yes**	Main text: CT examination and image preprocessing; Model construction; Supplement Appendix S1	Preprocessing includes CT resampling, window setting, ROI resizing, ImageNet normalization, z-score standardization, and LASSO feature selection.
METHODS - Data	**10**	Selection of data subsets	**Yes**	Main text: CT examination and image preprocessing; Supplement Appendix S1	Each patient contributed one L3 VAT image, one L3 SM image, and one 3D pancreatic ROI; L3 selection and ROI inputs are described.
METHODS - Data	**11**	De-identification methods	**Yes**	Supplement Appendix S1: Data handling	All imaging and clinical data were de-identified before analysis.
METHODS - Data	**12**	How missing data were handled	**Yes**	Main text: Patients; Supplement Appendix S1: Data handling	No imputation was performed; incomplete clinical data and incomplete/poor-quality CT images

					were excluded.
METHODS - Data	**13**	Image acquisition protocol	**Yes**	Supplement Appendix S1: CT examination and image preprocessing	Scanner manufacturers, scan range, tube voltage/current modulation, collimation, pitch, rotation time, matrix, slice thickness, and interval are reported.
METHODS - Reference Standard	**14**	Definition of method(s) used to obtain reference standard	**Yes**	Main text: Patient grouping criteria; Supplement Appendix S1	AP diagnosis and severity labels follow established diagnostic criteria and the 2012 revised Atlanta classification.
METHODS - Reference Standard	**15**	Rationale for choosing the reference standard	**Yes**	Main text: Patient grouping criteria; Supplement Appendix S1	The 2012 revised Atlanta classification is used as the standard clinical severity classification for AP.
METHODS - Reference Standard	**16**	Source of reference standard annotations	**Yes**	Supplement Appendix S1: Patient grouping criteria; Main text: CT preprocessing	Severity classification was determined from clinical records, laboratory data, imaging findings, and organ failure documentation; ROI annotations involved radiologists.
METHODS - Reference Standard	**17**	Annotation of test set	**Yes**	Main text: CT examination and image preprocessing; Supplement Appendix S1	The same severity criteria were applied across partitions; pancreas was delineated in ITK-SNAP 3.8 and VAT/SM were segmented with an

					open-source tool followed by manual correction.
METHODS - Reference Standard	**18**	Measures of inter- and intrarater variability of features described by the annotators	**Yes**	Main text: Reproducibility assessment; Supplement Table S1	Inter- and intra-observer reproducibility was assessed in 30 cases using DSC, HD95, and ASD.
METHODS - Data Partitions	**19**	How data were assigned to partitions	**Yes**	Main text: Patients; Supplement Appendix S1: Data partitioning	Center 1 was randomly split 7:3 into training and internal test sets; all eligible Center 2 cases formed the external test set.
METHODS - Data Partitions	**20**	Level at which partitions are disjoint	**Yes**	Supplement Appendix S1: Data partitioning	Partitions were disjoint at the patient level, with no patient overlap among training, internal test, and external test sets.
METHODS - Testing Data	**21**	Intended sample size	**Yes**	Main text: Statistical analysis; Supplement Appendix S1: Sample size considerations	A minimum sample size of approximately 284 patients was estimated; the final study population was 536 patients.
METHODS - Model	**22**	Detailed description of model	**Yes**	Main text: 2D and 3D DL model development; Model construction; Supplement Appendix S1; Figure 2	2D ResNet18 body-composition models, 3D ResNet18 pancreatic model, DL feature extraction, LASSO, and model fusion are described.
METHODS - Model	**23**	Software libraries, frameworks, and	**Yes**	Main text: CT preprocessing;	Reported software includes

		packages		Statistical analysis; Supplement Appendix S1	OnekeyAI platform version 6.3.15 for DL model development, ITK-SNAP version 3.8 for pancreas delineation, and R version 4.3.0 for statistics.
METHODS - Model	**24**	Initialization of model parameters	**Yes**	Main text: 2D and 3D DL model development; Supplement Appendix S1	2D ResNet18 used ImageNet-pretrained weights and transfer learning; the pancreatic model used a pretrained 3D ResNet18 architecture and was trained on the training set.
METHODS - Training	**25**	Details of training approach	**Yes**	Supplement Appendix S1: 2D and 3D DL model development and feature extraction	Augmentation, optimizers, learning rates, batch sizes, epochs, weight decay, predefined hyperparameters, and lack of a separate tuning subset are reported.
METHODS - Training	**26**	Method of selecting the final model	**Yes**	Supplement Appendix S1; Supplement Tables S8-S9; Main text: Results	The combined model was prespecified as the primary model based on the study objective
METHODS - Training	**27**	Ensembling techniques	**NA**	Main text: Model construction	The final approach is a feature/clinical fusion model rather than an ensemble of multiple model outputs.
METHODS - Evaluation	**28**	Metrics of model performance	**Yes**	Main text: Model comparison; Table	Metrics include AUC, accuracy,

				3; Supplement Table S5; Figures 3-4	sensitivity, specificity, PPV, NPV, F1, calibration, DCA, NRI, and IDI.
METHODS - Evaluation	**29**	Statistical measures of significance and uncertainty	**Yes**	Main text: Model comparison; Statistical analysis; Tables 2-3; Supplement Tables S2-S5	The manuscript reports 95% CIs, DeLong tests, p values, NRI/IDI CIs, and R version 4.3.0.
METHODS - Evaluation	**30**	Robustness or sensitivity analysis	**Yes**	Main text: Results; Supplement Figure S1-S3; Supplement Tables S8-S9	Subgroup analyses, SAP versus non-SAP analysis, and exploratory architecture comparisons are reported.
METHODS - Evaluation	**31**	Methods for explainability or interpretability	**Yes**	Main text: Model construction; Model interpretability; Figure 5	Grad-CAM and SHAP were used for model interpretation and feature contribution visualization.
METHODS - Evaluation	**32**	Evaluation on internal data	**Yes**	Main text: Patients; Results; Table 3; Figures 3-4	The internal test set included 128 Center 1 patients and is reported with AUC and classification metrics.
METHODS - Evaluation	**33**	Testing on external data	**Yes**	Main text: Patients; Results; Table 3; Supplement Appendix S1	The external test set included 110 Center 2 patients and is reported with performance metrics and comparisons.
METHODS - Evaluation	**34**	Clinical trial registration	**NA**	Main text: Patients	The study is retrospective and observational, not a clinical trial.
RESULTS - Data	**35**	Numbers of patients or examinations included and excluded	**Yes**	Main text: Results, Baseline characteristics; Figure 1	The manuscript reports 709 screened, 173 excluded by reason,

					and 536 final patients with partition sizes.
RESULTS - Data	**36**	Demographic and clinical characteristics of cases in each partition and dataset	**Yes**	Main text: Results; Table 1	Table 1 reports demographics and clinical variables across training, internal test, and external test sets.
RESULTS - Model Performance	**37**	Performance metrics and measures of statistical uncertainty	**Yes**	Main text: Results; Table 3; Supplement Tables S2-S5	AUCs with 95% CIs and full classification metrics are reported for all models and datasets.
RESULTS - Model Performance	**38**	Estimates of diagnostic performance and their precision	**Yes**	Main text: Results; Table 3; Figures 3-4; Supplement Table S5	ROC analysis, 95% CIs, calibration, DCA, and predictive clinical variables are reported.
RESULTS - Model Performance	**39**	Failure analysis of incorrectly classified cases	**Yes**	Supplement Table S6	Confusion matrices are provided for the combined model in the training, internal test, and external test sets, with TN/FP/FN/TP counts.
DISCUSSION	**40**	Study limitations	**Yes**	Main text: Discussion	Limitations include retrospective design, same-country centers, L3-only VAT/SM assessment, manual pancreas segmentation, and partial explainability.
DISCUSSION	**41**	Implications for practice, including intended use and/or clinical role	**Yes**	Main text: Discussion; Conclusion	The manuscript discusses early AP risk stratification using routine CT and the need for future prospective

					multicenter validation and automated segmentation.
OTHER INFORMATION	**42**	Provide a reference to the full study protocol or to additional technical details	**Yes**	Supplement Appendix S1; Supplement Tables S1-S9; Supplement Figures S1-S3	Additional technical details are provided in the supplement, including preprocessing, training settings, sample size, comparisons, and subgroup analyses.
OTHER INFORMATION	**43**	Statement about the availability of software, trained model, and/or data	**Yes**	Declarations: Availability of data and material	The datasets are stated to be available from the corresponding author on reasonable request
OTHER INFORMATION	**44**	Sources of funding and other support; role of funders	**Yes**	Declarations: Funding; journal submission system	Funding source is reported in the Declarations section, and the role of the funder has been declared in the journal submission workflow according to journal requirements.

References

1. Xu W, Fu Y-L, Zhu D (2023) ResNet and its application to medical image processing: Research progress and challenges. Computer Methods and Programs in Biomedicine 240:107660. https://doi.org/10.1016/j.cmpb.2023.107660

2. Xu G, Wang X, Wu X, et al (2025) Development of residual learning in deep neural networks for computer vision: A survey. Engineering Applications of Artificial Intelligence 142:109890. https://doi.org/10.1016/j.engappai.2024.109890

3. Shen Y, Guo P, Wu J, et al (2024) MoViT: Memorizing vision transformers for medical image analysis. In: Cao X, Xu X, Rekik I, et al (eds) Machine Learning in Medical Imaging. Springer Nature Switzerland, Cham, pp 205–213

4. Chen Z, Wang Y, Zhang H, et al (2023) Deep learning models for severity prediction of acute pancreatitis in the early phase from abdominal nonenhanced computed tomography images. Pancreas 52:e45–e53. https://doi.org/10.1097/MPA.0000000000002216

5. Liang H, Wang M, Wen Y, et al (2023) Predicting acute pancreatitis severity with enhanced computed tomography scans using convolutional neural networks. Sci Rep 13:17514. https://doi.org/10.1038/s41598-023-44828-7

6. Yin M, Lin J, Wang Y, et al (2024) Development and validation of a multimodal model in predicting severe acute pancreatitis based on radiomics and deep learning. International Journal of Medical Informatics 184:105341. https://doi.org/10.1016/j.ijmedinf.2024.105341

7. Xu Y, Teutsch B, Zeng W, et al (2026) Deep learning-based prediction of acute pancreatitis severity from abdominal CT with multicenter external validation. Radiology Advances 3:umag020. https://doi.org/10.1093/radadv/umag020
